# Recovery pattern after decompression of central lumbar spinal stenosis: a prospective observational cohort study

**DOI:** 10.1186/s13018-024-04614-1

**Published:** 2024-03-25

**Authors:** Niyaz Hareni, Soheil Ebrahimnia, Björn E. Rosengren, Magnus K. Karlsson

**Affiliations:** 1 Departments of Clinical Sciences and Orthopaedics, Lund University, Skåne University Hospital, Malmö, Sweden; 2 Department of Orthopaedics, Varberg Hospital, Träslövsvägen 68, 432 37 Varberg, Sweden

**Keywords:** Back, Decompression, Leg, Lumbar, Pain, Quality of life, Spinal, Stenosis, Surgery

## Abstract

**Background:**

Detailed preoperative information is associated with superior outcomes. We aimed to describe the recovery pattern after decompression of central lumbar spinal stenosis (CLSS).

**Methods:**

50 patients aged 51–85 years who underwent decompression without fusion due to CLSS were followed from before to after surgery (post-op day 1, 7, and 14). Back and leg pain were evaluated using the Numeric Rating Scale (NRS; 0 = no pain 0, 10 = worst pain) and quality of life using the EuroQol-5D index (0 = death, 1 = best), and EQ-5D-visual analogue scale (VAS; 0 = worst, 100 = best).

**Results:**

NRS leg pain was reduced from preoperative to first postoperative day by 5.2 (6.1, 4.3) (mean (95%CI)], and NRS back pain from postoperative day 1–7 by 0.6 (1.2, 0.03) and from day 7 to 14 by 0.7 (1.3, 0.2)]. In contrast, EQ-5D index increased from preoperative to first postoperative day by 0.09 (0.06, 0.13) and from day 1 to 7 by 0.05 (0.02,0.08), and EQ-5D VAS from preoperative to first postoperative day by 13.7 (9.1, 18.3) and from day 1 to 7 by 6.0 (2.0, 10.0). After two weeks, 51% of the patients had improved above the minimal clinically important difference (MCID) in back pain and 71% in leg pain.

**Conclusions:**

Patients scheduled for decompression due to CLSS should be informed that improvement in leg pain and quality of life in general can be expected within one day of surgery, that quality of life improves a little further in the first postoperative week, and that back pain improves in the first 2 postoperative weeks. In most patients, decompression without fusion due to CLSS seems to achieve clinically relevant improvement within 2 weeks.

## Background

There is no generally accepted definition of central lumbar spinal stenosis (CLSS), i.e. spinal canal reduction, and/or this type of anatomic abnormalities. Several different classifications are available [1, 2], with no gold standard [3]. This makes it difficult to compare results from different studies and cohorts [3]. However, when using a sagittal canal diameter of ≤ 10 mm as the definition of absolute CLSS, the prevalence has been reported to be 4% in individuals aged < 40 years and 20% in ages 60–69 years [1]. When using ≤ 12 mm as a relative/absolute CLSS, the prevalence was 20% and 47% in the two above age groups [1].

To complicate matters, a radiographically identified CLSS may occur with or without clinical symptoms. Neurogenic claudication is regarded as the most typical symptom, usually precipitated by prolonged standing and/or walking, and relived by sitting and/or bending forward [4, 5]. The pathophysiology behind neurogenic claudication is usually referred to bulging discs, osteoarthritis of the facet joints, and/or infolding of the ligamentum flavum, resulting in a mechanical compression of neural elements [6, 7] and/or ischaemia of lumbosacral nerve roots [6, 7]. CLSS may or may not also be accompanied by back pain [4, 5]. Previous studies have found no clear association between narrowing of the spinal canal and clinical symptoms [3, 8]. Finally, CLSS morbidity is often described as being associated with marked loss of independence and decreased quality of life [9].

Most CLSS patients in need of intervention are given patient education, physiotherapy, pain medication and/or epidural injections [10, 11]. One reason is that CLSS symptoms are often mild and fluctuating [7, 12]. With more persisting disability that does not respond to non-surgical treatment, decompressive surgery is regarded as the gold standard treatment [10, 13–15]. Randomised controlled trials (RCT) have found superior outcomes with decompressive surgery compared to non-operative treatment [14, 16, 17], with similar outcomes with and without fusion [13, 15]. Presently, it is not clear if certain subgroups of CLSS patients may in fact attain better outcome through an accompanying fusion [18].

The mid- and long-term outcomes after CLSS surgery have been evaluated [10, 13–18]. When conducting such evaluations, it is important to not only evaluate statistically significant differences, but also whether improvements exceed the minimal clinically important differences (MCID) [19]. To our knowledge, no study has evaluated recovery patterns after CLSS surgery in the immediate postoperative period. This is important, as improved knowledge about the postoperative period may make patient information more accurate. Currently, 54% of patients with degenerative lumbar spine surgery report that they are dissatisfied with the preoperative information [20]. Inadequate information is associated with inferior surgical outcomes and less satisfied patients [21–23]. Improved knowledge regarding the recovery pattern after CLSS surgery would therefore not only provide realistic expectations and improved ability to optimise aftercare planning and pain medication, but possibly also contribute to more satisfied patients.

The primary aims of this study were to identify (1) if decompression due to CLSS surgery within the first 2 postoperative weeks is associated with reduced pain and improved quality of life, (2) if the recovery pattern in back and/or leg pain and quality of life differ, (3) if there are sex differences, and (4) if improvement exceeds minimal clinical important difference.

## Material and method

From March 2020 to January 2022, we invited fifty patients scheduled for CLSS surgery at Ängelholm County Hospital, Sweden, due to symptoms not responding to non-surgical treatment. This hospital only conducts spinal surgery in patients in ASA (American Society of Anaesthesiologists) class 1 or 2. In order to be asked to participate in our study, the patients had to have 1–4 levels of CLSS confirmed by magnetic resonance imaging (MRI) [2], be scheduled for CLSS decompression surgery without fusion, and be over age 50 without cognitive impairment and with sufficient knowledge of Swedish to complete the questionnaire. All invited patients accepted, 25 men with a mean age of 66 years (range 51–82) and 25 women with a mean age of 72 years (range 55–85). Forty-six of the patients had both neurogenic claudication and back pain, 3 had only neurogenic claudication and one only back pain. Orthopaedic spine surgeons from Skåne University Hospital (SUS), Malmö, Sweden, performed the surgical procedures.

We retrieved pre- and perioperative data from SweSpine (the Swedish National Registry for Spine Surgery) [24–26]. The registry collects patient-reported data on age, sex, smoking habits, self-estimated walking distance in four categories [(1) < 100 m (m), (2) 100–500 m, (3) 500–1000 m, (4) > 1000 m], consumption of analgesics in three categories [(1) no use, (2) intermittent use or (3) continuous use], and use of opioid-related compounds. SweSpine also includes patient-reported outcome scores (PROMs), i.e. level of leg and back pain according to Numeric Rating Scale (NRS; 0 = no pain, 10 = worst imaginable pain) and quality of life according to EuroQol 5D index (EQ-5D index; 1 = best possible quality of life, 0 = death, negative values indicate conditions “worse than death”) and EQ5D visual analogue scale (EQ-5D VAS; 100 = best possible quality of life, 0 = worst). The surgeon reports the perioperative data to SweSpine, including whether the surgery was acute or elective, diagnosis, type of surgical procedure, level(s) of surgery, surgery-related complications, e.g. dural tear or injury to nerve roots and postoperative complications during the hospital stay through dichotomous answers (yes/no) on questions regarding death, postoperative haematoma, urinary retention, urinary tract infection, pulmonary embolism, wound infection, Cauda Equina Syndrome, thrombosis or “other complications”.

Two independent observers, uninvolved in the treatment of the patients, undertook interviews for this specific study on day 1, 7 and 14 after the operation. The observers then registered NRS back and NRS leg pain, EQ-5D index, EQ-5D VAS and pain medication. We regarded an improvement of ≥ 40% in NRS leg pain and of ≥ 33% in NRS back pain as clinically important differences based on previous research on minimal clinically important differences (MCID) [19].

We used Statistica version 12 (Stat Soft®) for all statistical calculations. Descriptive data are reported as means ± standard deviations (SD), means (ranges) or proportions (%). Inferential statistics are reported as means with 95% confidence interval (95% CI). Group comparisons for continuous variables were done using the Student unpaired t test, for repeated measurements with Student paired t test and analyses of variance (ANOVA), and for categorical variables using McNemar’s test. A *p* value of < 0.05 was regarded as a statistically significant difference. All patients gave written consent. The study was approved by the ethics committee in Lund, Sweden (EPN Dnr 2016/159).

## Results

**Fig. 1:**
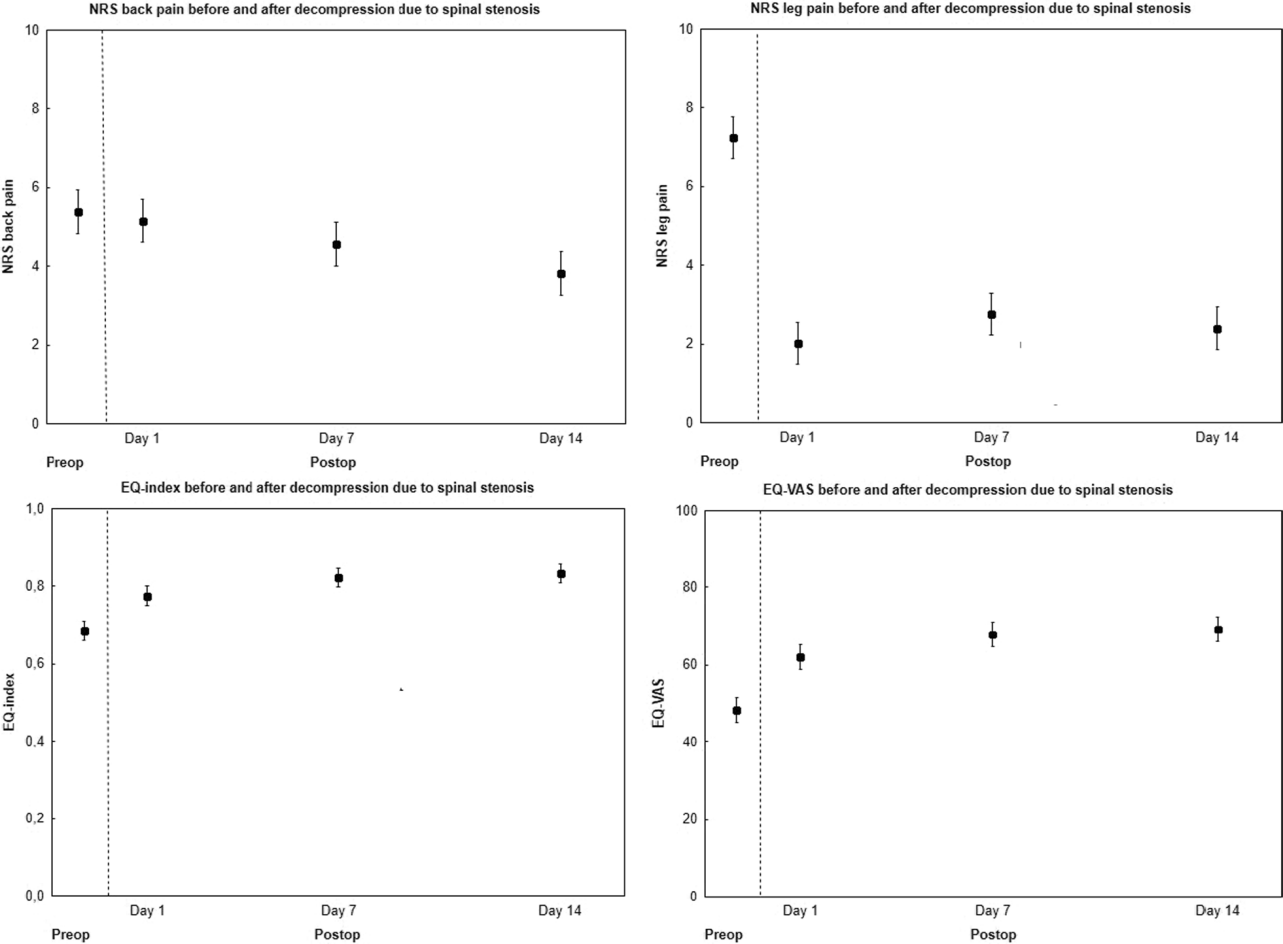
Patient-reported back and leg pain (Numeric Rating Scale; NRS) quality of life [EQ-5D index and EQ-5D VAS (visual analogue scale] before and 1, 7, and 14 days after surgery in 50 patients aged 51–85 years who underwent decompression due to central lumbar spinal stenosis (CLSS). Data are shown as mean with 95% confidence intervals (95% CI)

Table 1 shows pre- and perioperative patient characteristics. Table 2 and Fig. 1 show NRS back and leg pain preoperative, day 1, 7, and 14 after the operation. Table 3 and Fig. 1 show the EQ-5D index and EQ-5D VAS. Table 4 shows analgesic use. Seven patients were on continuous morphine/opioid opioanalogue medication preoperatively; one day after surgery the figure was 48 patients, after 7 days 33 patients, and after 14 days 17 patients.

**Table 1:** Preoperative data on age, smoking, back and leg pain, analgesics, walking distances and peri- and postoperative complications in 50 patients aged 51–85 years who underwent decompression due to central lumbar spinal stenosis (CLSS)

	Men (*n* = 25)	Women (*n* = 25)
Anthropometry		
Age (years)	66 ± 8	71 ± 9
Height (cm)	178 ± 7	164 ± 6
Weight (kg)	88 ± 12	74 ± 10
Body mass index (kg/m^2^)	28 ± 4	28 ± 4
Smokers [*n*]	2	1
Duration of back pain [*n*]		
No back pain	1	3
< 3 months	1	0
3 to < 12 months	4	1
12 to < 24 months	3	6
24 months or more	14	14
Missing data	2	1
Duration of leg pain [*n*]		
No leg pain	1	1
< 3 months	0	0
3 to < 12 months	5	3
12– 24 months	2	6
24 months or more	16	15
Missing data	1	0
Estimated walking distance [*n*]		
< 100 m	6	7
100–< 500 m	13	7
500–< 1000 m	4	7
1000 m or more	2	3
Missing data	0	1
Type of operation [*n*]		
Laminectomy without microscope	25	25
Number of operated level(s) [*n*]		
One level	15	17
Two levels	7	8
Three levels	2	0
> Three levels	1	0
Level(s) of surgery [*n*]		
L4–L5	10	9
L3–L4	4	6
L3–L5	7	7
Other level(s)	4	3
Complication [*n*]		
Dural rifts	2	1
Nerve root injury/cauda equine syndrome	0	0
Others	3	2

Data are presented as means ± standard deviations (SD) or numbers

**Table 2:** Patient-reported back and leg pain (Numeric Rating Scale; NRS) before and 1, 7, and 14 days after surgery in 50 patients aged 51–85 years who underwent decompression due to central lumbar spinal stenosis (CLSS)

	Preoperative	Postoperative			*p* value	
	Day 1	Day 1	Day 7	Day 14	Preoperative vs. postop day 1	Between postoperative days
NRS back pain						
All (*n* = 50)	5.4 (4.6, 6.1)	5.2 (4.5, 5.9)	4.6 (3.9, 5.2)	3.8 (3.1, 4.5)	0.61	**0.02**
Men (*n* = 25)	5.4 (4.4, 6.5)	4.8 (3.8, 5.9)	4.0 (3.1, 4.9)	3.5 (2.5, 4.6)	0.37	0.17
Women (*n* = 25)	5.3 (4.1, 6.5)	5.5 (4.5, 6.4)	5.1 (4.2, 6.0)	4.1 (3.1, 5.1)	0.78	0.10
*p *value (men vs. women)	0.87	0.36	0.09	0.39	–	–
NRS leg pain						
All (*n* = 50)	7.2 (6.7, 7.8)	2.0 (1.3, 2.7)	2.8 (2.0, 3.5)	2.4 (1.7, 3.1)	** < 0.001**	0.33
Men (*n* = 25)	7.0 (6.3, 7.8)	1.6 (0.6, 2.5)	2.1 (1.1, 3.1)	1.8 (0.8, 2.8)	** < 0.001**	0.75
Women (*n* = 25)	7.4 (6.6, 8.3)	2.5 (1.5, 3.5)	3.4 (2.4, 4.5)	3.0 (1.9, 4.0)	** < 0.001**	0.40
*p* value (men vs. women)	0.48	0.17	0.06	0.12	–	–

Data are presented as means (95% CI). Differences between preoperative to postop day 1 were tested using paired Student’s t test, between postoperative days using repeated measure analyses of variance (ANOVA) and between men and women using unpaired Student’s t test. Statistically significant p values are bolded

**Table 3:** Patient-reported quality of life [EQ-5D index and EQ-5D VAS (visual analogue scale] before and 1, 7, and 14 days after surgery in 50 patients aged 51–85 years who underwent decompression due to central lumbar spinal stenosis (CLSS). Data are presented as means (95% CI)

	Preoperative	Postoperative			*p* value	
	Day 1	Day 1	Day 7	Day 14	Preoperative vs. postop day 1	Between postoperative days
EQ-5D index						
All (*n* = 50)	0.68 (0.66, 0.71)	0.78 (0.74, 0.81)	0.82 (0.79, 0.86)	0.83 (0.81, 0.86)	**< 0.001**	**0.02**
Men (*n* = 25)	0.68 (0.64, 0.72)	0.78 (0.73, 0.83)	0.82 (0.77, 0.88)	0.85 (0.80, 0.89)	**< 0.001**	0.16
Women (*n* = 25)	0.69 (0.65, 0.73)	0.77 (0.73, 0.81)	0.83 (0.78, 0.87)	0.82 (0.78, 0.86)	**0.002**	0.11
*p* value (men vs. women)	0.56	0.77	0.90	0.32	**–**	**–**
EQ-5D VAS						
All (*n* = 50)	48.2 (44.6, 51.9)	62.0 (57.7, 66.2)	67.9 (63.6, 72.3)	69.3 (65.5, 73.0)	**< 0.001**	**0.03**
Men (*n* = 25)	47.4 (41.9, 52.9)	63.1 (56.1, 70.1)	68.0 (61.3, 74.8)	71.6 (66.1, 77.1)	**< 0.001**	0.16
Women (*n* = 25)	49.1 (44.0, 54.2)	60.9 (55.5, 66.2)	67.8 (61.8, 73.9)	66.9 (62.4, 72.3)	**< 0.001**	0.15
*p* value (men vs. women)	0.64	0.61	0.96	0.21	**–**	**–**

Differences between preoperative to postop day 1 were tested using paired Student’s t test, between postoperative days using repeated measure analyses of variance (ANOVA) and between men and women using unpaired Student’s t test. Statistically significant p values are bolded

**Table 4:** Patient-reported analgesics intake before and 1, 7, and 14 days after surgery in 50 patients aged 51–85 years who underwent decompression due to central lumbar spinal stenosis (CLSS)

	Preoperative (*n* = 49)	Postoperative (*n* = 50)			*p* value	
		Day 1	Day 7	Day 14	Preoperative vs. postop day 1	Between postoperative day
No analgesics	5 (10%)	0	4 (8%)	9 (18%)	**< 0.001**	**< 0.001**
Intermittent analgesics	16 (33%)	0	4 (8%)	9 (18%)		
Regular analgesics	28 (57%)	50 (100%)	42 (84%)	32 (64%)		

Preoperative data are missing in one patient. Data are presented as numbers with proportions (%) in brackets. Differences between preoperative and postoperative days are tested with McNemar’s Test (*n* = 49). Statistically significant p values are highlighted in bold text

NRS back pain was similar between preoperative and day 1 after surgery, but then decreased, from day 1 to 7 after surgery [− 0.6 (− 1.2, − 0.03)] and from day 7 to 14 (− 0.7 (− 1.3, − 0.2)] (Table 5). NRS leg pain decreased from preoperative to day 1 after surgery [− 5.2 (− 6.1, − 4.3)], but thereafter remained stable (Table 5). The EQ-5D index improved from preoperative to day 1 after surgery [+ 0.09 (0.06, 0.13)] and from day 1 to day 7 after surgery [+ 0.05 (0.02, 0.08)], but thereafter remained stable (Table 5). The EQ-5D VAS improved from preoperative to day 1 after surgery [+ 13.7 (9.1, 18.3)] and from day 1 to day 7 after surgery [+ 6.0 (2.0, 10.0)], but then remained stable (Table 5).

**Table 5:** Patient-reported improvement in back and leg pain (Numeric Rating Scale; NRS) and quality of life [EQ-5D index and EQ-5D VAS (visual analogue scale] from before to after surgery, and during different postoperative periods in 50 patients aged 51–85 years who underwent decompression due to central lumbar spinal stenosis (CLSS)

Changes during different periods				
	Preoperative to postop day 1	Postop day 1 to postop day 7	Postop day 7 to postop day 14	Preoperative to postop day 14
NRS back pain				
All (*n* = 50)	− 0.2 (− 1.1, 0.6)	**− 0.6 (− 1.2, − 0.03)**	**− 0.7 (− 1.3, − 0.2)**	**− 1.6 (− 2.3, − 0.8)**
Men (*n* = 25)	− 0.6 (− 2.0, 0.8)	**− 0.8 (− 1.6, − 0.01)**	**− 0.5 (− 1.4, − 0.4)**	**− 1.9 (− 3.0, − 0.8)**
Women (*n* = 25)	0.2 (− 1.0, 1.3)	− 0.4 (− 1.3, 0.5)	**− 1.0 (− 1.6, − 0.3)**	**− 1.2 (− 2.2, − 0.2)**
NRS leg pain				
All (*n* = 50)	**− 5.2 (− 6.1, − 4.3)**	**0.7 (0.06, 1.4)**	− 0.4 (− 0.8, 0.1)	**− 4.8 (− 5.8, − 3.9)**
Men (*n* = 25)	**− 5.5 (− 6.7, − 4.2)**	0.5 (− 0.2, 1.3)	− 0.2 (− 0.9, 0.5)	**− 5.2 (− 6.6, − 3.8)**
Women (*n* = 25)	**− 5.0 (− 6.4, − 3.5)**	1.0 (− 0.2, 2.1)	− 0.5 (− 1.2, 0.2)	**− 4.5 (− 5.9, − 3.1)**
EQ5D index				
All (*n* = 50)	**0.09 (0.06, 0.13)**	**0.05 (0.02, 0.08)**	0.01 (− 0.02, 0.04)	**0.15 (0.12, 0.18)**
Men (*n* = 25)	**0.10 (0.05, 0.15)**	**0.04 (0.0, 0.09)**	0.03 (− 0.02, 0.07)	**0.17 (0.13, 0.21)**
Women (*n* = 25)	**0.08 (0.03, 0.13)**	**0.05 (0.01, 0.10)**	− 0.01 (− 0.03, 0.02)	**0.13 (0.09, 0.17)**
EQ5D VAS				
All (*n* = 50)	**13.7 (9.1, 18.3)**	**6.0 (2.0, 10.0)**	1.3 (− 1.9, 4.5)	**21.0 (17.0, 25.0)**
Men (*n* = 25)	**15.7 (8.8, 22.6)**	5.0 (− 0.5, 10.5)	3.6 (− 2.0, 9.2)	**24.2 (18.7, 29.7)**
Women (*n* = 25)	**11.8 (5.3, 18.2)**	**7.0 (0.8, 13.1)**	− 0.9 (− 4.2, 2.3)	**17.8 (11.9, 23.7)**

Data are presented as means (95% CI). Differences between preoperative to postop day 1 were tested using paired Student’s t test, between postoperative days using repeated measure analyses of variance (ANOVA) and between men and women using unpaired Student’s t test. Statistically significant p values are bolded

Two weeks after surgery, 51% of the patients had improved above the minimal clinically important difference (MCID) in back pain and 71% in leg pain (Table 6).

**Table 6:** Proportion of patients with reported improvement in back and leg pain (Numeric Rating Scale; NRS) exceeding the minimal clinically important difference (MCID) 1, 7, and 14 days after surgery in the 49 patients with preoperative leg pain, and in the 47 with preoperative back pain, aged 51–85 years who underwent decompression due to central lumbar spinal stenosis (CLSS)

Preoperative	Postoperative—proportion of patients with improvement > MCID		
	Day 1	Day 7	Day 14
NRS back pain 1–10 (*n* = 47)	11 (23%)	15 (32%)	24 (51%)
NRS leg pain 1–10 (*n* = 49)	40 (82%)	33 (67%)	35 (71%)

Patients with no preoperative back pain or no leg pain were not included in this analysis as they had no hypothetical possibility to be improved > MCID by surgery. Data are presented as numbers (*n*) with proportions (%) in brackets

We found no sex differences in NRS leg pain, NRS back pain, EQ-5D index, or EQ-5D VAS preoperative day 1, 7 or 14 after surgery (Tables 2, 3) and no apparent sex differences in improvement after surgery (Table 5).

## Discussion

Decompression due to CLSS is generally followed by reduction in leg pain and improvement in quality of life within the first day of surgery; quality of life improves further the first postoperative week, and back pain decreases the first postoperative weeks. It is also important to emphasise that surgery is not only associated with statistically significant improvement, but in the majority of the patients also improvement of a clinically important difference [19]. Our data supported that decompression without fusion in CLSS patient is within weeks associated with favourable outcomes in the majority of the patients [10, 13–15].

We found that the recovery patterns for back and leg pain differ, without apparent sex differences. Leg pain improved within a day of surgery, while back pain improved gradually during the first 2 postoperative weeks. A similar temporal recovery pattern in pain improvement has previously been reported after lumbar disc herniation surgery [27]. Therefore, we speculate that the reduction in leg pain is predominantly the result of instant mechanical decompression of the nerve roots, while reduction in back pain may be influenced by other pathophysiological pathways, such as gradual reduction in the chemical inflammation associated with bulging discs, nucleus pulposus, and spinal canal compression [28, 29]. The slower improvement in back pain may also reflect the surgical trauma. Our data also indicate the importance of both back and leg pain for quality of life, as the EQ-5D index and EQ-5D VAS improved both from before to the first postoperative day (corroborating with reduction in leg pain) and the first postoperative week (corroborating with reduction in back pain).

The lower NRS leg pain and higher quality of life in the immediate postoperative period in our cohort compared to the one-year outcome after this type of operation that has been reported with SweSpine data (VAS leg pain 34, EQ 5D index 0.63, EQ5D VAS 64) [25, 26] may have several explanations. The well-known placebo effect of surgical interventions may influence the immediate postoperative PROMs rating. The personal contact between the patients and the researchers during the weeks before and after the operation may also influence the PROMs ratings, as good communication and thorough information is associated with favourable outcomes [21–23]. We also speculate, as PROMs rating is entirely subjective, that the first weeks of improvement after a longstanding disability could be perceived by the patient as a marked difference, leading to a high PROMs scoring. If the patient then expects further improvement that does not occur, this may lead to lower PROMs rating at the one-year follow-up. It is also possible that new pathological conditions, e.g. scar tissue and/or bulging discs, deteriorate the clinical status during the first postoperative year, also resulting in lower PROMs ratings at the one-year follow-up compared to the immediate postoperative period. Finally, ageing may be another contributing factor, as higher age is associated with lower PROMs rating.

Study strengths include the use of validated PROMs, and the inclusion of several surgeons with different experience, mimicking the general health care system. Even if this study only includes a small sample, we are of the opinion that our inferences can be generalised, as the preoperative NRS leg and back pain and the age and sex distribution were similar to nationally reported data [26]. Another strength is that none of the participants were lost to follow-up. The use of independent observers, unaware of the expectations of the surgeon or patient, preoperative treatments, radiological findings, surgical procedure, level(s) of surgery and/or complications are other study strengths. Limitations include the small sample size mentioned above and also that the patients were not consecutively included, and that only patients with ASA grade 1 and 2 and patients that understood Swedish were included.

## Conclusions

We conclude that decompression without fusion due to CLSS is associated with improvement in leg pain and quality of life already within one day of surgery, that quality of life improves further during the first postoperative week, and that back pain gradually decreases during the first 2 postoperative weeks. Decompression without fusion due to CLSS seems to achieve improvement with a clinically relevant difference in a majority of patients within 2 weeks.

## Data Availability

The data are available upon ethical review and permission from the SweSpine board.

## Abbreviations

CLSS: Central lumbar spinal stenosis
MCID: Minimal clinical important difference
NRS: Numeric Rating Scale
EQ-5D: EuroQol-5D
RCT: Randomised Controlled Trial
